# Spontaneous Iliac Vein Ruptures: A Systematic Review

**DOI:** 10.1177/15385744231163707

**Published:** 2023-03-13

**Authors:** Alexander L. Kooiman, Jay M. Bakas, Joris M. K. van Fessem, Willem P.A. Boellaard, Sandra A.P. Cornelissen, Marie Josee E. van Rijn

**Affiliations:** 1 Department of Vascular Surgery, 273198 Erasmus Medical Center Rotterdam, Rotterdam, The Netherlands; 2 Department of Anesthesiology, 273198 Erasmus Medical Center Rotterdam, Rotterdam, The Netherlands; 3 Department of Urology, 273198 Erasmus Medical Center Rotterdam, Rotterdam, The Netherlands; 4 Department of Radiology and Nuclear Medicine, 273198 Erasmus Medical Center Rotterdam, Rotterdam, The Netherlands

**Keywords:** rupture, spontaneous, iliac vein, venous, thrombosis, may-thurner syndrome, hematoma, case reports

## Abstract

**Introduction:**

Spontaneous iliac vein rupture is a rare, but frequently lethal condition. It is important to timely recognize its clinical features and immediately start adequate treatment. We aimed to increase awareness to clinical features, specific diagnostics, and treatment strategies of spontaneous iliac vein rupture by evaluating the current literature.

**Methods:**

A systematic search was conducted in EMBASE, Ovid MEDLINE, Cochrane, Web of Science, and Google Scholar from inception until January 23, 2023, without any restrictions. Two reviewers independently screened for eligibility and selected studies describing a spontaneous iliac vein rupture. Patient characteristics, clinical features, diagnostics, treatment strategies, and survival outcomes were collected from included studies.

**Results:**

We included 76 cases (64 studies) from the literature, mostly presenting with left-sided spontaneous iliac vein rupture (96.1%). Patients were predominantly female (84.2%), had a mean age of 61 years, and frequently presented with a concomitant deep vein thrombosis (DVT) (84.2%). After various follow-up times, 77.6% of the patients survived, either after conservative, endovascular, or open treatment. Endovenous or hybrid procedures were frequently performed if the diagnose was made before treatment, and almost all survived. Open treatment was common if the venous rupture was missed, for some cases leading to death.

**Conclusion:**

Spontaneous iliac vein rupture is rare and easily missed. The diagnose should at least be considered for middle-aged and elderly females presenting with hemorrhagic shock and concomitant left-sided DVT. There are various treatment strategies for spontaneous iliac vein rupture. An early diagnose brings options for endovenous treatment, which seems to have good survival outcomes based on previously described cases.

## Introduction

Spontaneous causes of retroperitoneal bleeding are easily missed due to its rare entity and uncommon presentation.^1^ Spontaneous iliac vein bleeding is such a rare cause, with a poorly understood etiology. Patients mostly present with symptoms and/or signs of a deep vein thrombosis (DVT), but evidence is lacking to prove it as the actual cause of the bleeding.^2,3^ Compression of the left common iliac vein by the right common iliac artery, an anatomical variant called the May-Thurner syndrome (MTS), might explain why spontaneous iliac vein ruptures commonly affect the left side.^3^

The frequently deathly consequences of a spontaneous iliac vein rupture emphasize the need to raise awareness for the wide clinical presentation, understand its underlying etiology, and learn from previous clinical decisions. The most comprehensive literature review on spontaneous iliac vein rupture was published in 2006 and included 33 patients.^3^ Many cases of spontaneous iliac vein rupture have been published since and new endovenous treatment techniques have been developed. Improved knowledge on the etiology, clinical presentation, treatment and outcomes of spontaneous iliac vein rupture may help to timely recognize, diagnose, and treat these patients.

This systematic review aims to provide an overview of patient characteristics, etiology, diagnostics, and treatment modalities of spontaneous iliac vein rupture.

## Methods

### Protocol

The protocol for this systematic review was entered in the International Prospective Register of Systematic Reviews PROSPERO network (CRD42023392812), and conducted according to the PRISMA statement.^4^

### Literature Search

A systematic search was conducted in EMBASE, Ovid MEDLINE, Cochrane, Web of Science, and Google Scholar from inception until January 23, 2023, without any restrictions. A library scientist was consulted to develop the literature search. Terms such as “spontaneous”, “unexpected”, “non-traumatic”, “vein rupture”, “tear”, and “iliac vein” were included. The complete search strategy is shown in Appendix A.

### Study Selection

All English full-text available case reports and case series were reviewed after screening for title and abstract. All studies describing one or more cases of spontaneous iliac vein ruptures and its management were eligible for inclusion, including case-reports, case-series, and reviews. Non-spontaneous iliac vein ruptures, such as trauma, ruptured aneurysm, arteriovenous fistula, complications, and iatrogenic ruptures were excluded. Editorials, letters, comments, and abstract publications were also excluded. Ruptures of other veins than the iliac veins, such as femoral vein, collaterals, or inferior vena cava were also excluded.

The CARE checklist was considered to check the completeness of studies.^5^ References from included review articles were checked manually, and considered for additional inclusion if data was described sufficiently.

Two authors (AK, JB) independently screened studies for eligibility after duplicate removal. Articles were first screened by titles and abstracts, and full-text screened thereafter. Disagreements for study in- and exclusion were resolved by negotiation in which a third reviewer (MR) was involved. The main authors of the studies were contacted if full-text was missing, or data were described insufficient for collection. Study selection was visualized using PRISMA flow diagram for study inclusion.^4^

### Data Extraction and Statistics

Data on patient characteristics, clinical presentation, and procedural characteristics were extracted from the included studies and are shown as count with percentages, mean with standard deviation, or median with interquartile ranges. Date of publication and countries where cases came from were also extracted.

## Results

### Cases

**Figure 1.**
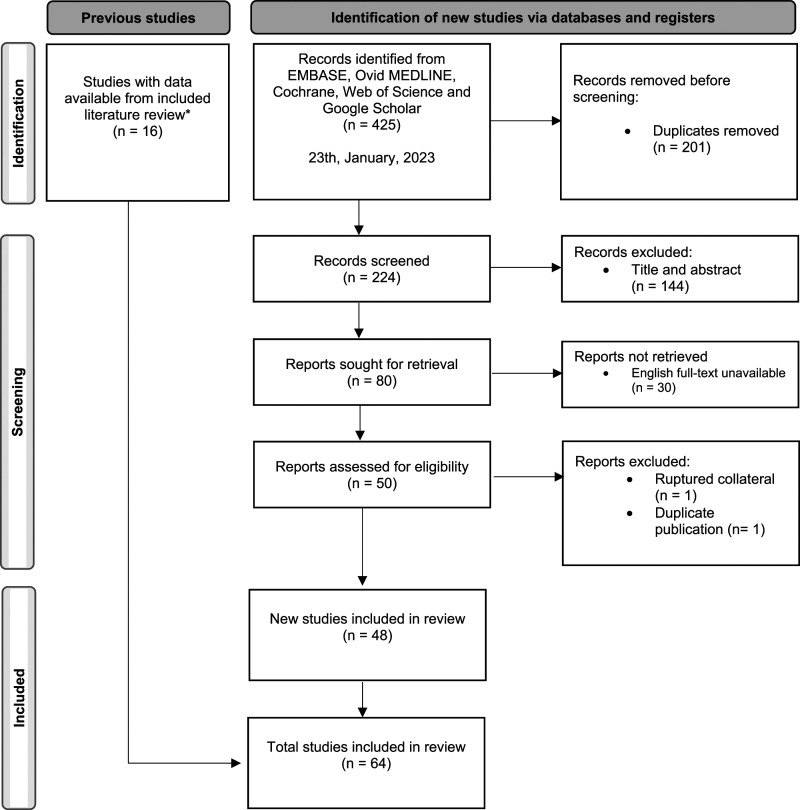
PRISMA flow diagram for systematic review study inclusion.

The literature search identified 224 articles after duplicate removal, the complete study selection is shown in Figure 1. Overall, 16 studies were included from a previous literature review. After full-text screening, 76 cases were included in this systematic review presented in 64 studies from all over the world (Table 1).^2,3,6-67^ Most patients underwent open surgery, the first reported in 1961. Endovenous procedures were reported since 2004. Patient and procedural characteristics are shown in Table 2. Patients were predominantly female (84.2%) with a mean age of 61 years at presentation. The bleeding was mainly located at the left side (96.1%). A concomitant DVT was reported in 84.2% of the patients. Abdominal pain was the most common complaint during initial presentation (82.9%), but leg swelling (53.9%), back pain (30.3%), and leg pain (31.6%) were also frequently reported, and 67.1% presented with signs of a hemorrhagic shock. Complaints started after physical activity (eg bending or walking), recent trauma (eg falling from height), defecation, and immobility. Constipation, obesity, previous pelvic surgery, and recent childbirth were also reported as preceding factors.

**Table 1.** Included studies after systematic literature search.

Study (n = 64)	Country	Case and Treatment (n = 76)
Hossne et al 1961* (6)	Brazil	n = 1, open
Herczeg et al1967* (7)	Germany	n = 1, open
Herrin et al 1975* (8)	USA	n = 1, open
Brown et al 1977 (9)	USA	n = 1, open
McDonald et al 1980* (10)	USA	n = 1, open
Elliot and Ware 1982 (11)	UK	n = 1, open
Estevan Solano et al 1982* (12)	Spain	n = 1, open
Noszczyk and Orezko 1983 (13)	Poland	n = 1, open
Stock and Gunn 1986 (14)	UK	n = 1, open
Forsberg et al 1988 (15)	Sweden	n = 2, open
Copeland et al 1989* (16)	UK	n = 1, open
Ratia et al 1989* (17)	Spain	n = 1, open
Hill et al 1990 (18)	UK	n = 1, open
Arikawa et al 1992 (19)	Japan	n = 1, open
van Damme et al 1993* (20)	Belgium	n = 1, open
Nishida et al 1993* (21)	Japan	n = 1, open
Majeed and Pheils 1993* (22)	UK	n = 1, open
McShannic et al 1994 (23)	USA	n = 1, open
Yamada et al 1995 (24)	Japan	n = 1, open
Plate and Qvarfordt 1995* (25)	Sweden	n = 1, open
Lin et al 1996 (26)	China	n = 1, open
DePass et al 1998 (27)	Canada	n = 1, open
Lopez-Boado et al 1998* (28)	Spain	n = 1, open
Gad et al 1999* (29)	Denmark	n = 1, open
Bracale et al 1999* (30)	Italy	n = 2, open
Gaschignard et al 2000 (31)	France	n = 1, open
Pedley et al 2002* (32)	UK	n = 1, open
Jazayeri et al 2002 (33)	France	n = 1, open
Tamura et al 2002 (34)	Japan	n = 1, conservative
Cho et al 2003 (35)	Korea	n = 1, conservative
Pavon et al 2003* (36)	Spain	n = 1, open
Kwon et al 2004 (37)	Korea	n = 1, open
Zieber et al 2004 (38)	USA	n = 1, endovascular
Yasuga et al 2004 (39)	Japan	n = 1, open
Tannous et al 2006 (3)	USA	n = 1, open
Kim et al 2007 (40)	Korea	n = 1, hybrid
Jiang et al 2010 (2)	China	n = 3, conservative
		n = 5, open
		n = 1, hybrid
Zang et al 2011 (41)	China	n = 1, open
Deng et al 2011 (42)	China	n = 1, open
Kim et al 2011 (43)	Korea	n = 1, open
Chen et al 2013 (44)	China	n = 1, open
Hughes et al 2013 (45)	USA	n = 1, endovascular
Sadaghianloo et al 2014 (46)	France	n = 1, open
Kim et al 2015 (47)	Korea	n = 1, conservative
Pattanshetti et al 2015 (48)	Indian	n = 1, conservative
Hosn et al 2016 (49)	USA	n = 1, hybrid
Kalender et al 2016 (50)	Turkey	n = 1, open
Joseph et al 2017 (51)	USA	n = 1, open
Chen et al 2018 (52)	Taiwan	n = 1, hybrid
Ingram et al 2019 (53)	USA	n = 1, endovascular Δ
Molière et al 2020 (54)	France	n = 1, conservative
Long et al 2020 (55)	USA	n = 1, conservative
De Campos Costa et al 2021 (56)	Portugal	n = 1, open
Sueyoshi et al 2021 (57)	Japan	n = 1, endovascular
Zheng et al 2021 (58)	China	n = 2, endovascular
McCready et al 2021 (59)	USA	n = 1, endovascular
		n = 1, open
Nishimoto et al 2021 (60)	Japan	n = 1, endovascular Δ
Ergüneş and Günerhan 2022 (61)	Turkey	n = 1, open
Eziolisa and Chapman 2022 (62)	USA	n = 1, open
Fugaretu et al 2022 (63)	Romania	n = 1, open
Glengarry et al 2022 (64)	Australia	n = 1, none
Qi et al 2022 (65)	China	n = 1, endovascular
Gruter and Oudkerk 2022 (66)	Netherlands	n = 1, hybrid
Huh et al 2022 (67)	Japan	n = 1, conservative

* = full-text missing, data extracted from Tannous et al; Δ = initially conservatively treated.

**Table 2.** Patient Characteristics, Presentation, Type of Treatment and Survival.

Patient Characteristics (n = 76)	Outcome
Age, years	61.4 ± 12.2
Female	64 (84.2)
Left-sided	73 (96.1)
**Presentation**	
Symptoms and signs	64 (84.2), concomitant deep vein thrombosis
	63 (82.9), abdominal pain
	51 (67.1), hemorrhagic shock
	41 (53.9), swollen leg
	24 (31.6), leg pain
	23 (30.3), back pain
	18 (23.7), abdominal mass
	6 (7.9), abdominal distension
	4 (5.3), leg ischemia
	4 (5.3), phlegmasia cerulea dolens
**Imaging**	
	46 (60.5), computed tomography
	18 (23.7), venography
	17 (22.4), ultrasound
	1 (1.3), magnetic resonance imaging
	9 (11.8), no imaging
**Type of intervention (n = 76)**	Survived
Conservative/none: n = 12*§*	9 (75.0)
Open procedures: n = 51	38 (74.5)
• Ligation: n = 13	
• Repair: n = 37	
• Hematoma removal: n = 1	
Endovascular procedures: n = 8	8 (100)
• Coiling: n = 2	
• Thrombectomy and stenting: n = 6	
Hybrid procedures: n = 5	4 (80.0)
• Stenting followed by laparotomy: n = 2	
• Open repair followed by stenting for MTS: n = 3	

Continuous variables presented as mean with standard deviation (SD); categorical variables presented as count (%);§ Thrombolysis started for concomitant DVT, massive bleeding from spontaneous iliac vein rupture, patient died before intervention was possible {Hughes, 2013 #13}. MTS = May-Thurner Syndrome.

### Treatment

Patients underwent either conservative, endovascular, hybrid, or open surgical repair (Table 2). Conservative treatment was suitable for hemodynamically stable patients, including in-hospital monitoring, followed by anticoagulant treatment in case of concomitant DVT. Open, endovascular, or hybrid surgical procedures were considered for patients with hypovolemic shock, suspicion of active bleeding, or as additional treatment for MTS. Open surgical procedures mostly included primary repair (n = 37, 72.5%), while endovascular treatment consisted of venous stenting or embolization.

### Imaging

**Figure 2.**
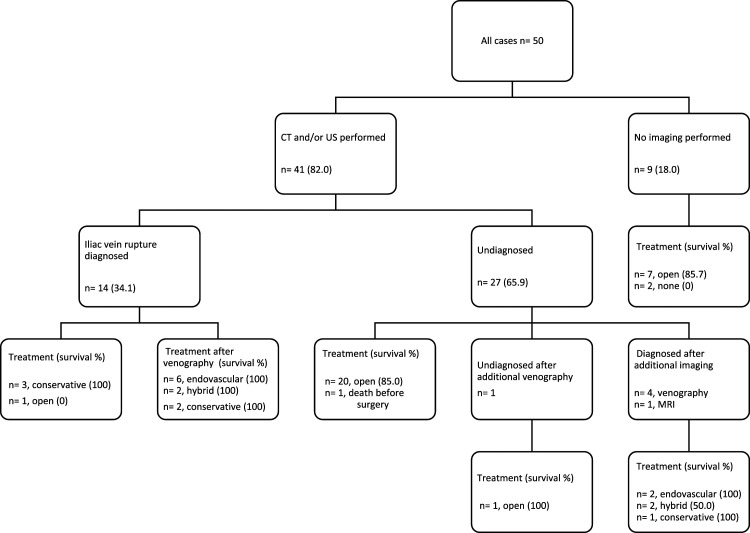
Diagnostics, treatment and survival. Data shown as count (%). A total of 26 cases were excluded from this flowchart because of missing data.

Data on imaging was available for 50 cases and are shown in Figure 2. In 18.0% (n = 9), no imaging was performed. After ultrasound and/or CT-imaging, an iliac vein rupture remained unrecognized in 65.9% (n = 27). In a subgroup of patients, additional imaging with venography or magnetic resonance imaging (MRI) was performed which then led to the correct diagnose, except for one case in which the rupture was also not diagnosed on the venography.^24^

#### Pre-operative Diagnosis and Treatment Strategy

In 31 cases (62.0%), the iliac vein rupture remained undiagnosed before start of treatment, even after imaging in some cases (Figure 2). These patients were all treated conservatively or with open surgery. In 18 of the 19 cases that were adequately identified as having an iliac vein rupture endovenous or hybrid interventions, or conservative treatment was performed. One case underwent open surgery after she suffered a cardiopulmonary arrest, just after the diagnose was made.^63^

### Survival

Overall, 59 patients (77.6%) survived the spontaneous iliac vein rupture and 17 patients died, shown in Table 2. Patients were at least followed until discharge with a maximum follow-up of 50 months. One patient survived the spontaneous iliac vein rupture, but died 7 weeks later of cardiac failure.^15^ After open surgery, 38 out of 51 patients survived, while all patients except for one, survived after endovenous or hybrid treatment. One patient died after stent placement, because she developed abdominal compartment syndrome due to the large retroperitoneal hematoma caused by the iliac vein rupture. Laparotomy for abdominal decompression and removal of ischemic intestines was of no avail.^66^

All conservatively treated patients survived except for 2 cases in which the rupture was missed and one case who died before reaching the hospital.^64^ One patient presenting with severe right lower back pain was misdiagnosed with lumbosacral strain and died from cardiac arrest after returning to the ER multiple times.^55^ Another patient received thrombolysis because of an extensive left-sided iliofemoral DVT. In the presence of an iliac vein rupture (conformed during autopsy) the patient started to bleed massively once the thrombus resolved and died before laparotomy could be performed.^45^

### Concomitant DVT Treatment

A concomitant DVT was reported in 84.2% of the cases, Table 2. Therapeutic anticoagulants were started for 44 out of 64 cases at various times after treatment. The timing ranged from admission, if the patient was hemodynamically stable, towards days after intervention. The duration of the anticoagulant therapy also differed between cases. Heparin, low molecular weight heparin, vitamin-K-antagonist and direct anticoagulants were prescribed. In two cases anticoagulants were not prescribed after conservative management despite a concomitant DVT.^34,35^ Pulmonary embolism was present in four cases.^14,47,64,67^

## Discussion

This is the first systematic literature review of spontaneous iliac vein rupture, describing 76 cases. Spontaneous iliac vein rupture mainly occurs in middle-aged or elderly women, is located predominantly on the left side, often with a concomitant DVT and over half of the cases present with signs of hemorrhagic shock. These ruptures often remain undiagnosed before a treatment strategy is chosen, even after imaging. Undiagnosed patients frequently undergo open surgery, while diagnosed patients are almost always treated with endovascular or hybrid repair.

The overrepresentation of case-reports and case-series in the current literature, emphasize the rare entity of the disease. Increased awareness of symptoms and signs of left-sided DVT, especially in middle-aged or elderly women, may help to early recognize spontaneous iliac vein rupture as the cause of hemorrhagic shock.

Various potential causes of spontaneous iliac vein rupture are hypothesized,^3^ but its actual etiology is poorly understood. Tannous et al. describe three potential etiologies of a spontaneous iliac vein rupture: mechanical, inflammatory, and hormonal.^3^ A combination of these factors is thought to explain spontaneous iliac vein rupture by increasing pressure, loss of vessel compliance and weakening of the vessel wall. A common presentation on the left side supports a mechanical cause as a result of compression of the left common iliac vein (MTS), while a concomitant DVT (found in 84%) leads to inflammation of the vessel. The finding that most patients were female and around 60 years-old may support a hormonal cause. While MTS was reported in only 21 patients within our review, the actual prevalence may have been underestimated, since left iliac vein compression is not routinely investigated and imaging was missing for several patients. Also, it is unclear whether the DVT is the cause or the result of the spontaneous iliac vein rupture as external compression from the retroperitoneal hematoma can provoke the DVT.

Timely recognition of spontaneous iliac vein rupture after radiological imaging gives the opportunity to consider endovascular treatment. Endovascular and hybrid procedures for spontaneous iliac vein bleeding reported promising survival rates. In case of concomitant DVT, start of anticoagulant therapy should be considered when the bleeding is stopped. The length of anticoagulant therapy should depend on the cause of DVT, either provoked or unprovoked.^68^ Due to publication bias, especially with case reports, we have to be careful to interpret these findings to optimistically, as less successful cases may remain unpublished. Selection bias may also have affected our findings. Patients that are hemodynamically stable may had more time to get diagnosed and set-up for endovascular interventions than unstable patients who went straight into the operating room. This most certainly will have affected survival outcomes.

## Conclusion

Spontaneous iliac vein rupture is rare, but frequently lethal and should be considered as a cause of hemorrhagic shock in patients presenting with a concomitant left-sided DVT, especially in middle-aged and elderly women. It is difficult to diagnose, even with the use of various imaging techniques. Multiple treatment methods are available including conservative, open, endovascular, or hybrid procedures. Timely identification gives the opportunity to consider endovascular treatment.

## Supplemental Material

Supplemental Material - Spontaneous Iliac Vein Ruptures: A Systematic Review Click here for additional data file. Supplemental Material for Spontaneous Iliac Vein Ruptures: A Systematic Review by Alexander L. Kooiman, BSc, Jay M. Bakas, MD, Joris M. K. van Fessem, MD, Willem P.A. Boellaard, MD, Sandra A.P. Cornelissen, MD, and Marie Josee E. van Rijn, MD, PhD in Vascular and Endovascular Surgery
