# Supplementary material for: Spontaneous Iliac Vein Ruptures: A Systematic Review
Source: Vasc Endovascular Surg. 2023 Mar 13;57(6):617–25. doi: 10.1177/15385744231163707 (PMC10291386; doi:10.1177/15385744231163707)
Supplement: Supplemental Material - Spontaneous Iliac Vein Ruptures: A Systematic Review [file sj-pdf-1-ves-10.1177_15385744231163707.pdf]

Spontaneous iliac vein rupture

Database searched	Platform	Years of coverage	Records	Records after duplicates removed
Medline ALL	Ovid	1946 - Present	90	89
Embase	Embase.com	1971 - Present	174	91
Web of Science Core Collection*	Web of Knowledge	1975 - Present	61	6
Cochrane Central Register of Controlled Trials	Wiley	1992 - Present	0	0
Additional Search Engines: Google Scholar*** (100 top-ranked)			100	45
**Total**			**425**	**231**

*Science Citation Index Expanded (1975-present) ; Social Sciences Citation Index (1975-present) ; Arts & Humanities Citation Index (1975-present) ; Conference Proceedings Citation Index- Science (1990-present) ; Conference Proceedings Citation Index- Social Science & Humanities (1990-present) ; Emerging Sources Citation Index (2005-present)
***Google Scholar was searched via "Publish or Perish" to download the results in EndNote.
No other database limits were used than those specified in the search strategies

Embase 174
((('vein rupture'/de OR rupture/de) AND ('iliac vein'/de OR 'external iliac vein'/de OR 'common iliac vein'/de)) OR (((spontan* OR idiopath* OR non-traum* OR nontraum* OR unknown* OR unexpect* OR non-iatrogen* OR noniatrogen*) NEAR/12 (iliac* OR ilio* OR femoroili*) NEAR/12 (vein* OR vena OR venous) NEAR/12 (ruptur* OR tear* OR break*))):ab,ti,kw)

Medline 90
((Rupture/ AND Iliac Vein/) OR (((spontan* OR idiopath* OR non-traum* OR nontraum* OR unknown* OR unexpect* OR non-iatrogen* OR noniatrogen*) ADJ12 (iliac* OR ilio* OR femoroili*) ADJ12 (vein* OR vena OR venous) ADJ12 (ruptur* OR tear* OR break*))).ab,ti,kf.)

Cochrane 0
((((spontan* OR idiopath* OR non-traum* OR nontraum* OR unknown* OR unexpect* OR non-iatrogen* OR noniatrogen*) NEAR/12 (iliac* OR ilio* OR femoroili*) NEAR/12 (vein* OR vena OR venous) NEAR/12 (ruptur* OR tear* OR break*))):ab,ti,kw)

Web of Science 61
TS=(((((spontan* OR idiopath* OR non-traum* OR nontraum* OR unknown* OR unexpect* OR non-iatrogen* OR noniatrogen*) NEAR/12 (iliac* OR ilio* OR femoroili*) NEAR/12 (vein* OR vena OR venous) NEAR/12 (ruptur* OR tear* OR break*)))))

Google Scholar
"spontaneous|idiopathic|unexpected iliac vein rupture"|"spontaneous|idiopathic|unexpected rupture**iliac vein"|"spontaneous|idiopathic|unexpected**iliac vein rupture"
'spontaneous|idiopathic|unexpected iliac vein rupture'|'spontaneous|idiopathic|unexpected rupture**iliac vein'|'spontaneous|idiopathic|unexpected**iliac vein rupture'
